# Patterns of early primary school-based literacy interventions among Pacific children from a nationwide health screening programme of 4 year olds

**DOI:** 10.1038/s41598-018-29939-w

**Published:** 2018-08-17

**Authors:** Philip J. Schluter, Jesse Kokaua, El-Shadan Tautolo, Rosalina Richards, Tufulasi Taleni, Hyun M. Kim, Richard Audas, Brigid McNeill, Barry Taylor, Gail Gillon

**Affiliations:** 1 University of Canterbury – Te Whare Wānanga o Waitaha, School of Health Sciences, Christchurch, New Zealand; 2 The University of Queensland, School of Clinical Medicine, Primary Care Clinical Unit, Brisbane, Australia; 3 University of Otago, Division of Health Sciences, Pacific Islands Research and Student Support Unit, Dunedin, New Zealand; 4 Auckland University of Technology, Centre for Pacific Health and Development Research, Auckland, New Zealand; 5 University of Otago, Dunedin School of Medicine, Department of the Dean, Dunedin, New Zealand; 6 University of Canterbury – Te Whare Wānanga o Waitaha, College of Education, Health and Human Development, Christchurch, New Zealand; 7 University of Otago, Dunedin School of Medicine, Department of Women and Children’s Health, Dunedin, New Zealand; 8 University of Canterbury – Te Whare Wānanga o Waitaha, School of Teacher Education, Christchurch, New Zealand

## Abstract

Literacy success is critical to unlocking a child’s potential and enhancing their future wellbeing. Thus, the early identification and redressing of literacy needs is vital. Pacific children have, on average, the lowest literacy achievement levels in New Zealand. However, this population is very diverse. This study sought to determine whether the current national health screening programme of pre-school children could be used as an early detection tool of Pacific children with the greatest literacy needs. Time-to-event analyses of literacy intervention data for Pacific children born in years 2005–2011 were employed. A multivariable Cox proportional hazard model was fitted, and predictive assessment made using training and test datasets. Overall, 59,760 Pacific children were included, with 6,861 (11.5%) receiving at least one literacy intervention. Tongan (hazard ratio [HR]: 1.33; 95% confidence interval [CI]: 1.23, 1.45) and Cook Island Māori (HR: 1.33; 95% CI: 1.21, 1.47) children were more likely to receive an intervention than Samoan children; whereas those children with both Pacific and non-Pacific ethnic identifications were less likely. However, the multivariable model lacked reasonable predictive power (Harrell’s *c*-statistic: 0.592; 95% CI: 0.583, 0.602). Regardless, important Pacific sub-populations emerged who would benefit from targeted literacy intervention or policy implementation.

## Introduction

It has long been recognised that early childhood literacy success is critical to a person’s health and wellbeing, enabling a realisation of his or her full potential. Moreover, it is perforce the engagement with and contribution to modern society^1^. Perhaps surprisingly the evidence-base for improving literacy in general populations has considerable gaps and challenges^2^, and so-called ‘hard-to-reach’ groups are often excluded^3^. Support of literacy is essential, and those with reading and learning needs should be identified early for intervention^4^. As literacy is such a basic foundational skill, delay in redressing these needs can have profound rippling effects over a child’s entire educational enterprise. Indeed, children requiring literacy interventions at primary school are already being ‘left-behind’. Emergent literacy skills (e.g., alphabet knowledge, print concepts, phonological awareness, and early writing) develop through sociocultural experiences from birth and are strong predictors of future reading and writing achievements^5,6^. Socioeconomic position and family practices also impact upon children’s early language and literacy development; two modes of communication that are deeply intertwined. For instance, in terms of language exposure and rate of expressive language development, socioeconomically advantaged children in a United States of America (USA) were exposed to and speaking over twice as many words as children from less advantaged families by age 3 years^7^. Thus, it is critical to detect literacy needs early so that timely remediation can be efficaciously applied.

Pacific people in New Zealand constitute a relatively young, fast growing immigrant ethnic minority, comprising 7% of the population with median age 22.1 years at the 2013 Census, compared to the dominant European population which comprised 74% of the population with median age 41.0 years^8^. Most Pacific people reside in Auckland, and they are generally less advantaged in income, education, housing, and health compared to their European counterparts^9,10^. As such, Pacific people are frequently overrepresented in many adverse health and social risk factors, leading to disproportionately higher health burden, premature death and consequent lower life expectancy^10^. Many of the causes are preventable and linked to social deprivation or poverty. Pacific children, many who are bilingual, are often left behind in the New Zealand school system. At primary school, on all available benchmarks, Pacific students achieve, on average, at lower levels in early English literacy and numeracy than all other students^11^. Here, English literacy has been defined by the dominant New Zealand European population and that this may differ from how literacy is understood in other cultures, including Pacific, which also emphasise other forms to the written symbol such as the spoken word, metaphors, proverbs, visuals arts, and song. Pacific students’ continued educational performance disparity compared to other ethnic groups in New Zealand has been labelled a ‘crisis’^12^, and is unacceptable to these communities and, indeed, the greater New Zealand society.

Quality early childhood programmes can help narrow the achievement gap between children from low-income families and those from more advantaged families^11^. Pacific primary school entrants have the lowest prior participation in early childhood education (ECE) services: 86.8% in 2012, compared to 90.9% of Māori entrants and 98% of European entrants^13^. Māori, New Zealand’s indigenous people, have been affected by colonisation and the ongoing effects of globalisation, and are also overrepresented in many adverse health and social risk factors. Increasing ECE participation for both Pacific and Māori children is a national priority. However, consultation with Pacific peoples suggest that early child centres are not, by themselves, a panacea, and could be improved by becoming more culturally inclusive^14^. Part of this inclusiveness would include broadening their value base to also include health and wellbeing in defining educational success^14^.

Despite typically labelled Pacific or Pasifika by educational and health agencies, the New Zealand Pacific population is heterogeneous made up of many ethnicities – manifest in differing cultures, languages, generations of immigrants, and the influences of acculturation^15^. There are over 20 Pacific nationalities resident in New Zealand; Samoans constituted the largest ethnic group (48.7%), followed by Cook Island Māori (20.9%), and Tongans (20.4%); and 62.3% were born in New Zealand^16^. Inter-ethnic relationships are common, both between Pacific nationalities and between Pacific and non-Pacific groups. Consistent with the recommended method of reporting in New Zealand, multiple ethnic identifications are permitted^17^. As such, in the 2013 Census, over 20% of all children aged 5–9 years were identified as belonging to two or more ethnic groups^18^. Therefore, Pacific communities are diverse and their health, education, and social needs are complex. However, this diversity of ethnic and cultural identities is lost under the homogeneous invoking single Pacific banner, and likely hides pockets of Pacific children who are doing relatively well and others who are doing relatively poorly. This behoves Pacific-specific investigations into factors associated with health and educational success and identifying those children who are at most risk and would benefit from timely and efficacious culturally appropriate interventions. Understanding these factors are critical for the appropriate targeted identification and intervention of individuals but also, more generally, to inform decision and policy-makers of subpopulation patterns that may most benefit from their attention. An opportunity for such an investigation exists using Statistics New Zealand – Tatauranga Aotearoa’s (SNZ’s) Integrated Data Infrastructure (IDI). The IDI captures national data collected from a range of government agencies, SNZ surveys (including the 2013 Census), and non-government organisations^19^. In particular, it houses data from both the Ministry of Health – Manatū Hauora (MoH) and the Ministry of Education – te Tāhuhu o te Mātauranga (MoE).

Within New Zealand, a nationwide screening programme of children aged 4 years was instigated in September 2008, and focuses on identifying any health, social, behavioural or development issues which could potentially interfere with children’s learning and success at school and to support children’s healthy development^20^. Known as the B4 School Check (B4SC), it includes measures of hearing and vision, height and weight, and behavioural and developmental questions using the Strengths and Difficulties Questionnaire (SDQ), completed separately by parents and ECE teachers, where applicable, and Parents’ Evaluation of Developmental Status (PEDS) tools^21^. Uptake is high, with 92% of the nation’s 4-year-old children participating in the 2015/2016 check^22^. Information collected as part of the B4SC is held by the MoH and available to the IDI. Additionally, the IDI contains important covariates, such as socio-demographic variables, including sex, month and year of birth, and a measure of deprivation.

The MoE has a stated purpose to lift aspirations, and raise educational achievement for every New Zealander^23^. As part of fulfilling this purpose, it seeks to provide school-based interventions to support children with identified behavioural or learning needs, and records each instance where a literacy intervention is provided to them. Student-level data on intervention type, date and timing, frequency and duration are also made available to the IDI. Linking B4SC data to this literacy intervention information thus enables an assessment of the B4SC’s utility as an early screen for predicting literacy intervention. This can be undertaken for the New Zealand population as a whole, as we have done^24^, but also for important specific groups, such as Pacific children. Reviews of early childhood development programmes demonstrate that such programmes can have a positive effect on children’s cognitive development and their readiness to succeed in their learning^25,26^. However, aside from readily identifiable infants and children with frank and apparent neurodevelopmental disorders, the general population screening of young children for future literacy need is recognised as being challenging^27–30^; due, in part, to the considerable variability in children’s emergent language and literacy development^28^. Although, few general population studies have had the suite of health variables available from within the B4SC screen.

When investigating a national sample, all considered socio-demographic and B4SC variables were significantly related to receiving a literacy intervention (p < 0.01)^24^. Boys were more likely to receive the intervention than girls, as were those living in higher deprivation. Large ethnic variations were observed with Māori children significantly more likely (hazard ratio [HR]: 1.34; 95% confidence interval [CI]: 1.27, 1.40) and Asian children significantly less likely (HR: 0.47; 95% CI: 0.43, 0.51) to receive a literacy intervention than their European counterparts. Children identified as being of Pacific ethnicity (HR: 1.33; 95% CI: 1.26, 1.41) had a similar likelihood of receiving an intervention as Māori children, but those identified with both Pacific and European ethnicities (HR: 1.00; 95% CI: 0.90, 1.10) had a significantly lower likelihood, despite children with any Pacific ethnic identification performing relatively worse on literacy and numeracy benchmarks.

In a large national population study, using comprehensive and psychometrically robust standardized health measures and education indicators, linked on the individual level by the internationally novel IDI, the primary aim is to provide contemporaneous quantitative insights into the relation between Pacific children’s health and development status at age 4 years and their early literacy development. Specifically, the study has two primary aims: (1) to investigate the factors captured within the B4SC health screen of Pacific children aged 4 years and report their association with literacy intervention in early primary-school, and (2) to ascertain whether these factors may be used as an early detection tool of those Pacific children with the greatest literacy need.

## Results

Over the study period, 475,965 children with valid date of births between 1 January 2005 and 31 December 2011 were captured within the IDI databases. Of these, 71,199 (36.0%) were identified as being of Pacific ethnicity. Level II ethnic identification information was available for 64,695 (90.9%) of these Pacific children. Restricting this IDI dataset to those enrolled with the MoE yielded 59,760 (83.9%) children of Pacific ethnicity, leaving 11,442 (16.1%) unmatched – through unsuccessful MoE linking, home-schooling, or emigration outside of New Zealand. These 59,760 children form the research database.

### Demographics

Table 1 presents the demographic distributions for Pacific children enrolled and contained within the MoE database and for those within the greater IDI database. From Table 1 it can been seen that there existed many different ethnic identification make-ups, with 38,118 (53.5%) having Pacific only ethnic identifications and 29,196 (41.0%) having combinations of Pacific, Māori and European identifications, the vast majority living in urban centres, and nearly 60% lived in the most deprived areas of New Zealand. Among the children grouped under the ‘Other Pacific’ ethnic grouping: 3,909 (37.8%) children had only level I ethnic information available (i.e. categorised broadly as Pacific); 2,499 (24.2%) identified with at least one Pacific ethnicity outside of the Samoan, Tongan and Cook Island Māori groups; 1,002 (9.7%) identified as being Samoan and at least one other Pacific ethnicity apart from Tongan or Cook Island Māori ethnic groups; 1,008 (9.7%) identified with both Samoan and Tongan ethnicities; 825 (8.0%) identified with both Samoan and Cook Island Māori ethnicities; and, 369 (4.6%) identified with both Tongan and Cook Island Māori ethnicities. Similarly, among the children grouped as ‘Pacific and Other’, 1,287 (40.8%) identified with Pacific and Asian ethnicities; 582 (18.5%) identified with Pacific, European and Asian ethnicities; 498 (15.8%) identified with Pacific, Māori, European and Asian ethnicities; and, 282 (8.9%) identified with Pacific, Māori and Asian ethnicities. When comparing the MoE sample with the IDI database, small but significant differences were observed between ethnic identification and deprivation level (both χ^2^ tests, p < 0.001) but not for sex (Fisher’s exact test, p = 0.40) or domicile area (Fisher’s exact test, p = 0.08). Samoan and other Pacific children and those residing in the most deprived areas were less likely to be in MoE sample than the IDI database, whereas children identified as being of Pacific and European or Pacific, Māori and European ethnicities were more likely to be in the MoE sample.

**Table 1:** Distribution of selected demographic variables for the full IDI dataset, and those eligible for this study (MoE linked sample).

	MoE sample n^a^ (%)	IDI dataset n^a^ (%)
*Ethnicity*		
Samoan	11,205 (18.8)	13,845 (19.4)
Cook Island Māori	3,342 (5.6)	3,948 (5.5)
Tongan	6,528 (10.9)	7,533 (10.6)
Other Pacific^b^	10,341 (17.3)	12,792 (18.0)
Pacific and Māori	7,782 (13.0)	9,201 (12.9)
Pacific and European	9,000 (15.1)	10,287 (14.4)
Pacific, Māori, and European	8,406 (14.1)	9,708 (13.6)
Pacific and Other	3,156 (5.3)	3,885 (5.5)
*Sex*		
Female	29,112 (48.7)	34,737 (48.8)
Male	30,648 (51.3)	36,465 (51.2)
*Domicile area* ^c^		
Urban	57,801 (97.0)	68,799 (97.1)
Rural	1,770 (3.0)	2,076 (2.9)
*Deprivation* ^d^		
Q1 (least deprived)	2,778 (4.6)	3,195 (4.5)
Q2	4,239 (7.1)	4,935 (6.9)
Q3	6,696 (11.2)	7,737 (10.9)
Q4	12,186 (20.4)	14,232 (20.0)
Q5 (most deprived)	33,642 (56.3)	40,740 (57.2)

Note: ^a^Randomly rounded to base 3, as per the confidentiality rules of SNZ. ^b^Includes children with level II information who were not Samoan, Cook Island Māori, or Tongan; children with two or more Pacific ethnic identifications; and, Pacific children with only level I information available. ^c^Data missing for 327 (0.5%) in the IDI dataset. ^d^Data missing for 363 (0.5%) in the IDI dataset.

Multiple Pacific ethnicity identities were common. Within the IDI database, 8,985 (12.6%) children identified with at least two, 927 (1.3%) with at least three, and 24 (0.03%) with at least four Pacific ethnicities. These patterns were similar in the MoE sample.

### Literacy intervention

**Figure 1:**
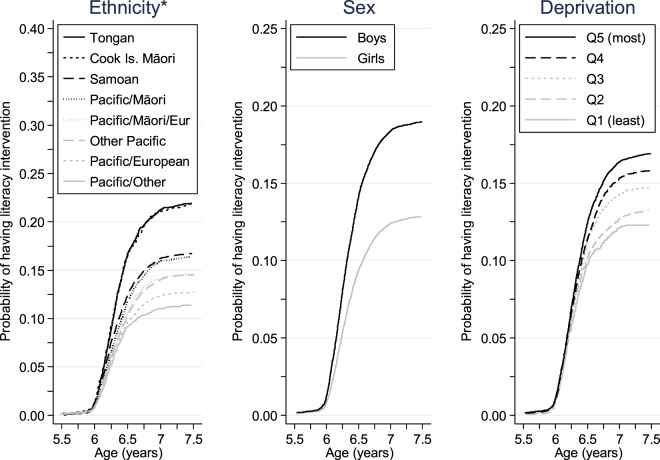
Kaplan-Meier curves of probability of having a literacy intervention by ethnicity, sex and deprivation, measured using quintiles of the NZDep2013 score (note: *the vertical axis for ethnicity is different from that used for sex and deprivation).

By 31 December 2015, 6,861 (11.5%) children had received at least one literacy intervention. Since birth, the children’s median participation time in the study was 7.1 years (Q_1_ = 6.0, Q_3_ = 9.0 years), and the median age to children’s first literacy intervention was 6.3 years (Q_1_ = 6.1, Q_3_ = 6.5 years). Figure 1 depicts the Kaplan-Meier curves for the probability of literacy intervention for demographic variables ethnicity, sex and deprivation. This figure illustrates that children’s first literacy intervention typically occurred between 6 and 7 years of age, and substantial differences appeared within ethnic, sex and deprivation groups.

Table 2 presents the numbers (%) of children who had at least one literacy intervention by these selected demographics, together with unadjusted and adjusted hazard ratios HRs and associated 95% CIs derived from Cox’s proportional hazard model. All of the demographics investigated were significantly related to literacy intervention in both unadjusted and adjusted models (all p < 0.001). In particular, Tongan and Cook Island Māori children, males, those resident in a rural area, and increasing deprivation were all associated with an increased likelihood of receiving an intervention compared to their reference counterparts. Based on this model, a Tongan boy living in a rural area within the highest NZDep2013 quintile would have a literacy intervention HR of 2.86 (95% CI: 2.30, 3.57) compared to an urban living Samoan girl within the least deprived area – assuming their B4SC profiles were identical.

**Table 2:** Numbers (%) of B4SC cohort who had at least one literacy intervention by selected demographics, together with unadjusted and adjusted hazard ratios (HRs) and associated 95% confidence intervals (CIs).

	N^a^	Literacy interventionn^a^ (%)	Unadjusted HR (95% CI)	Adjusted^b^ HR (95% CI)
*Ethnicity*				
Samoan	11,205	1,398 (12.5)	1 (reference)	1 (reference)
Cook Island Māori	3,345	534 (16.0)	1.34 (1.22, 1.49)	1.33 (1.21, 1.47)
Tongan	6,528	1,005 (15.4)	1.34 (1.23, 1.45)	1.33 (1.23, 1.45)
Other Pacific^c^	10,341	1,107 (10.7)	0.86 (0.79, 0.93)	0.85 (0.78, 0.92)
Pacific/Māori	7,779	903 (11.6)	0.98 (0.90, 1.06)	0.96 (0.88, 1.05)
Pacific/European	9,000	813 (9.0)	0.74 (0.68, 0.81)	0.80 (0.73, 0.87)
Pacific/Māori/European	8406	852 (10.1)	0.86 (0.79, 0.94)	0.90 (0.82, 0.98)
Pacific/Other	3,156	255 (8.1)	0.66 (0.58, 0.76)	0.68 (0.59, 0.78)
*Sex*				
Female	29,112	2,676 (9.2)	1 (reference)	1 (reference)
Male	30,648	4,182 (13.6)	1.54 (1.46, 1.61)	1.53 (1.46, 1.61)
*Domicile area*				
Urban	57,801	6,639 (11.5)	1 (reference)	1 (reference)
Rural	1,770	201 (11.4)	1.04 (0.90, 1.19)	1.18 (1.03, 1.37)
*Deprivation*				
Q1 (least deprived)	2,778	246 (8.9)	1 (reference)	1 (reference)
Q2	4,239	396 (9.3)	1.08 (0.92, 1.27)	1.03 (0.88, 1.21)
Q3	6,696	708 (10.6)	1.22 (1.05, 1.41)	1.13 (0.97, 1.30)
Q4	12,183	1,386 (11.4)	1.31 (1.14, 1.50)	1.16 (1.01, 1.33)
Q5 (most deprived)	33,645	4,098 (12.2)	1.40 (1.23, 1.59)	1.18 (1.04, 1.35)
*Hearing*				
Pass	31,533	2,841 (9.0)	1 (reference)	1 (reference)
Pass after rescreen	4,953	723 (14.6)	1.53 (1.41, 1.66)	1.41 (1.29, 1.53)
Fail - referral	4,311	504 (11.7)	1.24 (1.13, 1.36)	1.15 (1.04, 1.26)
Under care	1,077	90 (8.4)	1.03 (0.83, 1.27)	0.94 (0.75, 1.18)
Declined test	2,964	525 (17.7)	1.42 (1.29, 1.56)	1.39 (1.27, 1.53)^d^
Not done (missing)	14,922	2,181 (14.6)	1.24 (1.18, 1.31)	1.21 (1.11, 1.33)^d^
*Vision*				
Pass	35,256	3,321 (9.4)	1 (reference)	1 (reference)
Pass after rescreen	2,496	375 (15.0)	1.51 (1.36, 1.68)	1.33 (1.18, 1.49)
Fail - referral	3,477	429 (12.3)	1.28 (1.16, 1.42)	1.25 (1.13, 1.39)
Under care	711	51 (7.2)	0.77 (0.58, 1.02)	0.81 (0.60, 1.08)
Declined test	2,889	513 (17.8)	1.35 (1.23, 1.49)	1.38 (1.25, 1.52)^d^
Not done (missing)	14,934	2,178 (14.6)	1.19 (1.13, 1.26)	1.21 (1.11, 1.32)^d^
*BMI categories*				
Normal/underweight	17,994	1,707 (9.5)	1 (reference)	1 (reference)
Overweight	8,895	885 (9.9)	1.06 (0.98, 1.15)	1.01 (0.93, 1.09)
Obese	8,985	963 (10.7)	1.13 (1.04, 1.22)	1.03 (0.95, 1.11)
Morbidly obese	3,576	411 (11.5)	1.17 (1.05, 1.30)	1.03 (0.92, 1.15)
Not done (missing)	20,307	2,901 (14.3)	1.16 (1.09, 1.23)	1.07 (0.90, 1.28)
*PEDS*				
No action	31,224	3,273 (10.5)	1 (reference)	1 (reference)
Advice given	5,931	495 (8.3)	1.20 (1.09, 1.31)	1.15 (1.03, 1.29)
Referred	1,887	234 (12.4)	1.53 (1.34, 1.75)	1.37 (1.18, 1.59)
Referred – declined	246	15 (6.1)	1.23 (0.76, 2.00)	1.25 (0.76, 2.05)
Under care	930	60 (6.5)	1.15 (0.89, 1.48)	1.19 (0.87, 1.60)
Declined test	375	33 (8.8)	0.81 (0.58, 1.13)	0.64 (0.43, 0.95)
Not done (missing)	19,173	2,757 (14.4)	1.13 (1.08, 1.19)	1.07 (0.89, 1.29)^d^
*SDQ-parent/caregiver*				
No action	32,646	3,417 (10.5)	1 (reference)	1 (reference)
Advice given	5,361	432 (8.1)	1.13 (1.03, 1.25)	0.95 (0.84, 1.08)
Referred	1,050	117 (11.1)	1.47 (1.23, 1.77)	1.12 (0.91, 1.38)
Referred – declined	408	21 (5.1)	1.01 (0.67, 1.54)	0.90 (0.59, 1.37)
Under care	432	27 (6.3)	1.04 (0.71, 1.54)	0.93 (0.60, 1.46)
Declined test	687	96 (14.0)	1.21 (0.99, 1.49)	1.15 (0.91, 1.46)
Not done (missing)	19,176	2,754 (14.4)	1.12 (1.06, 1.17)	1.02 (0.84, 1.23)^d^
*SDQ-teacher*				
No action	12,942	1,254 (9.7)	1 (reference)	1 (reference)
Advice given	657	78 (11.9)	1.46 (1.16, 1.84)	1.26 (0.99, 1.61)
Referred	315	33 (10.5)	1.18 (0.84, 1.67)	0.94 (0.66, 1.34)
Referred – declined	72	6 (8.3)	0.96 (0.40, 2.32)	0.81 (0.33, 1.98)
Under care	150	9 (6.0)	0.88 (0.43, 1.78)	0.74 (0.35, 1.53)
Declined test	12,084	1,194 (9.9)	1.21 (1.12, 1.31)	1.14 (1.05, 1.23)
Not done (missing)	19,212	2,760 (14.4)	1.16 (1.08, 1.24)	1.01 (0.83, 1.21)^d^
Not applicable	14,334	1,530 (10.7)	1.01 (0.94, 1.09)	0.91 (0.84, 0.98)

Note: ^a^Randomly rounded to base 3, as per the confidentiality rules of SNZ. ^b^Adjusted for all variables listed in Table 3. ^c^Includes children with level II information who were not Samoan, Cook Island Māori, or Tongan; children with two or more Pacific ethnic identifications; and, Pacific children with only level I information available. ^d^Estimates determined by omitting collinear indicator variables.

### B4SC measures

Of the 59,760 Pacific children within the MoE research database, 49,512 (82.9%) were indicated as being contacted for a B4SC. Significant ethnic differences were observed in B4SC participant within the sample (χ^2^ test, p < 0.001) with inclusion rates ranging from 65.8% for Other Pacific and 81.1% for Pacific and Other children through to 89.2% for Pacific and European and 89.6% for Pacific and Māori and European children. B4SC measurement status showed that 30,096 (60.8%) children had their checks completed and their file closed, 10,749 (21.7%) had checks completed but their status remained open, 7,467 (15.1%) were partially completed and were assigned for completion, and 1,203 (2.4%) were returned without completion. The median age B4SC measures were undertaken was 4.3 years (Q_1_ = 4.1, Q_3_ = 4.6 years). Also included in Table 2 is the empirical distribution of the sample with literacy intervention by the considered B4SC variables, together with their crude and adjusted HR estimates.

In the unadjusted analyses, all considered B4SC variables were significantly related to the likelihood of receiving a literacy intervention (all p < 0.001). Excluding those children with not applicable SDQ–teacher assessments, the overall agreement between SDQ–parent/caregiver and SDQ–teacher categories was slight, with κ = 0.093 (95% CI: 0.087, 0.0.098) and 12,120 (46.2%) children having concordant outcomes. The primary discordance arose from the high rate of teachers declining to undertake the assessment; see Table 2. When investigating agreement between parents/caregiver and teacher SDQ assessments limited to those with “no action”, “advice given”, “referred”, and “referred – declined” outcomes for both assessments (n = 13,788), agreement was fair κ = 0.303 (95% CI: 0.282, 0.323) and concordance higher (84.7%). Discordance was greatest with parents/caregivers assessed as needing to have advice given whereas teachers’ SDQ assessment required no action.

In the multivariable model, which included all the demographic and B4SC variables investigated here, all demographic and B4SC variables remained significantly associated with this literacy intervention variable (all p < 0.001, apart from: domicile area p = 0.02 and deprivation p = 0.02), except for BMI (p = 0.91) and SDQ-parent/caregiver (p = 0.71); see Table 2. For hearing, the estimated risk for an intervention was significantly higher among those Pacific children who failed their first screen but past after rescreening (HR = 1.41) and for those who declined the test (HR = 1.39) compared to those who passed at their first screen. A similar pattern was observed for the vision screen, where the estimated risk for an intervention among those Pacific children who failed their first screen but past after rescreening (HR = 1.33) and for those who declined the test (HR = 1.38) was significantly higher than those who passed at their first screen. Compared to children with no detected developmental or behaviour problems, as measured by the PEDS, those who were referred (HR = 1.37) or where advice was given (HR = 1.15) had a significantly greater likelihood of receiving a literacy intervention. The estimated effect size was relatively large for those where a suggested referral was declined by the parents (HR = 1.25) after the PEDS assessment, but the relationship was non-significant (p = 0.38) due to the small numbers in this category. With respect to the SDQ in the multivariable model, the only category significantly associated with an increased risk of literacy intervention was where the teacher declined the test (HR = 1.14) compared to children where no action was indicated. This was despite the SDQ for both parents/caregivers and teachers having relative large and significant relationships with literacy intervention in the unadjusted analyses. Taken together, estimates from this model reveal that those children who pass their vision and hearing tests after rescreening will have a literacy intervention HR of 1.87 (95% CI: 1.66, 2.10) compared to those who pass both their initial tests, and if these children also were referred after the PEDS then their HR would further increase to 2.57 (95% CI: 2.13, 3.10) if this referral was accepted or 2.33 (95% CI: 1.40, 3.88) if it was declined.

The predictive power of the multivariable model, which included all demographic and B4SC variables investigated here, fitted on a training dataset and calculated on the remaining partitioned data, yielded a Harrell’s *c*-statistic of 0.592 (95% CI: 0.583, 0.602). According to the interpretation guidelines of Hosmer and Lemeshow, this represents better than chance prediction but less than the threshold (of 0.7) for a model demonstrating ‘reasonable’ predictive power. When considering the demographic variables alone in a multivariable, the estimated *c*-statistic was 0.580 (95% CI: 0.571, 0.590), significantly less than the full model (p < 0.001).

## Discussion

Large ethnic differences existed in the likelihood of receiving literacy interventions in early primary school years, with Tongan (HR: 1.33; 95% CI: 1.23, 1.45) and Cook Island Māori (HR: 1.33; 95% CI: 1.21, 1.47) children more likely to receive an intervention than Samoan children in the adjusted analyses; whereas those children with both Pacific and non-Pacific ethnic identifications having less likelihood of receiving a literacy intervention. The mechanism for these differences is unknown, although likely reflects variations in immigration histories and cultural practices^15^. Differential bilingualism profiles between Tongan and Samoan children and their households may provide one partial explanation. According to the New Zealand 2013 Census^31^, 51.1% and 86.3% of Tongan people speak Tongan and English fluently, respectively, whereas 61.8% and 87.4% of Samoan people speak Samoan and English fluently. This implies that significantly more Samoan people are bilingual than their Tongan counterparts. In addition to children’s emotional and behavioural benefits conferred by being bilingual^32^, there is evidence that academic developmental trajectories during their early school years is also improved compared to their monolingual peers – although this relationship is complex^33^. This may also partially explain the decreased likelihood of children with both Pacific and non-Pacific ethnic identifications receiving an intervention, as these children are more likely to be multilingual. In the same Census^31^, 93.6% of Cook Island Māori people reported speaking fluent English. In addition to English, Cook Islands Māori is an official language of the Cook Islands but has no official status in New Zealand, and data are not systematically collected. Additionally, Tongan and Cook Island Māori mothers in New Zealand are more likely than Samoan mothers to have both relatively low Pacific and European cultural orientations^15^. Retaining strong Pacific cultural links has been shown to have positive health benefits in New Zealand^15^, and it could be opined that such strong links would also lead to better educational outcomes for children from outside the prevailing European culture.

In addition to ethnicity, large and significant differences were observed between many of the considered demographic variables. Males, those living in rural areas, and those domicile in areas of increasing deprivation were all more likely to require and have experience with a literary intervention, consistent with reports elsewhere within New Zealand and overseas^24,34^. While the existence of a persistent sex differential in literacy achievement has been universally reported^34,35^, including among Pacific children living within New Zealand^24,36^, its causes, consequences, and potential solutions remain contested in both the public and professional discourse^34^. However, three key focus areas have been identified in the literature that helps explain gender gaps in literacy attainment: factors in school; in the home and community; and, in peer culture^35^. The challenge remains on how to address these factors in children’s early years. The findings in relation to deprivation were unsurprising^5,11^, but the extent and impact of this inequality was revealing. Nearly 60% of Pacific children were found to living in the most deprived areas of New Zealand and approximately 20% lived in the next most deprived quintile. National NZDep2013 figures for children aged 0–5 years at the 2013 Census revealed that 18.2%, 18.5%, 19.0%, 19.7%, and 24.6% lived in Q1 (least deprived) through to Q5 (most deprived) areas, respectively^37^. Those children growing up in the most deprived areas are more likely to be living in households with low income, material hardship and household crowding, resulting in reduced opportunities for health and educational provision and successes^38,39^.

In terms of the B4SC measures, hearing, vision, and developmental or behaviour problems measure were predictably and importantly related to literacy intervention likelihood, but BMI and SDQ were not (except when teachers declined the SDQ test). While significant in the unadjusted analysis, the effect associated with BMI was explained by other variables, as has been observed elsewhere^40^. The relationship between childhood obesity and academic achievement remains contested^41^. For the SDQ, this multi-informant instrument is one of the most widely used screening measure of emotional and behavioural health problems in children and youth but questions have been raised about its applicability within community samples^42^. Certainly, its utility in predicting literacy intervention among Pacific children was found wanting here. In terms of the significant variables, Pacific children with hearing and vision rescreens and referrals, and those who were referred from the PEDS were at significantly increased risk of a literacy intervention. Indeed, children who pass their vision and hearing tests after rescreening and were referred after the PEDS then had a HR for a literacy intervention estimated to be 2.57 (95% CI: 2.13, 3.10) if this referral was accepted or 2.33 (95% CI: 1.40, 3.88) if it was declined compared to children passed vision and hearing tests and having no action required after their PEDS assessment.

The increased likelihood of a literacy intervention for children who initially failed a hearing or vision screen but passed a subsequent rescreen is novel and warrants further investigation. This result may have been influenced by a range of factors, including that some children may have been false negatives to these hearing or vision tests. That is a child who passes the rescreen when they do in fact have an impairment. False negative results are of concern, as children who pass the screen are not routinely followed-up and likely to be at greater likelihood of falling behind in literacy and language. Few population studies report false negative rate information, and none were found for school entry hearing screen tests – although in localised hearing studies of children over disparate age ranges, false negative rates varied from 0.2% to 20%^43^. False negative rates and their impact are difficult to quantify. If a child passed the rescreen and was subsequently found to have an impairment, there can never be any certainty that the impairment was present on the day the child was screened. It could have been acquired or have progressed beyond an identifiable screen threshold level following the original screen. Alternatively, the impairment may be transient which, by its very nature, may not have been present at the time of rescreening. The two-staged approach to the B4SC vision and hearing tests is designed to reduce the likelihood of false negative results, but it cannot eliminate them – especially when undertaken on a national scale.

Another noteworthy feature of these analyses were the large number of declined hearing and vision assessments, together with the assessment not done (missing), and their association with increased literacy intervention likelihood. Access, cultural acceptability of services, racism, language - including barriers for interpreter service utilisation, and the use of alternative health care providers are likely to be just some of the myriad of factors that explain this finding^44^. The implication is that Pacific people are significantly less likely to ever attend primary health care than the dominant European or indigenous Māori people, and those who do access care generally attend at significantly lower comparative rates^44^. Moreover, this is likely to contribute to relatively poor follow-up rates. Findings from the Pacific Islands Families Study, a birth cohort of 1,398 Pacific children born in 2000 in South Auckland New Zealand^45,46^, revealed many Pacific children aged 2 years had middle ear disease. Of the 656 children who had confirmatory testing, 25.4% had otitis media with effusion (OME) in at least one ear and 7.5% had other otological abnormalities^47^. However, only 53% of children with possible OME or other abnormalities were in a position to attend an otolaryngological follow-up, despite it being offered locally and free of charge.

While PEDS is one commonly employed tool that examines parental concern regarding children’s cognition, communication, motor skills, and readiness for school^48^, studies that have examined the utility of the PEDS in predicting later literacy or academic success are relatively scarce and have produced mixed findings^49,50^. It is of note here that it was the Pacific children who were referred which were at significantly higher risk of a literacy intervention. This may result from the children failing to follow-up with the appropriate professional, that the professional failed to (or could only partially) attend to the child’s problems as they pertained to literacy achievement, or the individual or sociocultural circumstances were such that the referred children were more likely to remained disadvantaged. Further research is needed to further elucidate the mechanism(s) of this finding.

In terms of whether the B4SC factors may be used as an early detection tool of those Pacific children with the greatest literacy need, despite the constellation of significant risk factors, the multivariable model had only modest predictive abilities. The estimated *c*-statistic of 0.592 (95% CI: 0.583, 0.602) fell short of the threshold for a model to be considered as having reasonable predictive power^51^. Although terms such as risk factors and predictors are often conflated and used interchangeably within the epidemiological literature, they represent different concepts, and even strong associations do not necessarily indicate importantly increased predictive ability^52^. A more nuanced successful predictive model will likely need to be composed of a much broader suite of individual, family, and community-based variables, including those which elicit questions and capture tasks which are more explicitly focused on language and literacy development and need. For instance, in a study that examined the role that bilingualism played in children’s academic developmental trajectories during their early school years found that school-level factors explained about one third of the reductions in the differences^33^. It also emphasises the need and importance of longitudinal studies in providing information around the context and development of children that routine collected IDI data, however sophisticated, is unlikely to provide^45,46^. Lastly, it may also be the case that predicting early literacy need with high sensitivity and specificity from general population screening programmes is simply not possible^27–30^ due, in part, to the inherent variability in children’s emergent language and literacy development^28^.

While the study has salient strengths, it also suffers from some weaknesses. The principal strength is its investigation into a large recent sample of Pacific children - a population rarely studied. It also bring together variables not explored before on a population level, using linked inter-agency national datasets. However, on the flip-side, arguably the greatest potential weakness of this study is the measure of literacy used herein. The literacy intervention variable was constructed by bringing together ‘Reading recovery’ and ‘Specialist teaching’ indications, and thus its psychometric properties are unknown. Reading recovery is given to children with the lowest reading levels at each school. Thus, children who do not meet a particular level or difficulty threshold with literacy to receive the intervention but are identified as struggling in comparison to their peers. This means that the provision of literacy services are not equally distributed across the population, and even when offered, the standard for whether a child receives services is unlikely to be uniform between schools. Given that Pacific children are more likely to attend lower decile schools, what may look like a favourable outcome at age 6 years (i.e., no literacy intervention) may not be that favourable in terms of literacy achievement and, indeed, represent a hidden unmet need. As such, literacy intervention need is likely to be underestimated for Pacific children.

A further important limitation concerns the coverage and uptake rates of the B4SC over the study period. Although introduced in 2008, uptake rates in Auckland, where almost two-thirds (65.9%) of those who identified with at least one Pacific ethnicity lived^8^, was initially relatively low – increasing from approximately 35% in 2010/2011 to approximately 95% in 2015/2016^22,24^. If Auckland children have or are subjected to a different pattern of behaviour, then this may introduce non-sampling biases into the findings. However, analysis of information on B4SC attendance by area level deprivation between 2010/2011 and 2015/2015 revealed that uptake rates were similar for those living in deprived and non-deprived areas, hence the sample does not appear to be selective by deprivation^22^. In the national sample, 84.5% of children had their checks completed and their file closed^24^, compared to the 60.8% of Pacific children observed here. While this rate has increased from the reported 38.8% of Pacific children with checks completed and file closed born between July 2005 and January 2007^53^, many Pacific children are paradoxically missing or having incomplete assessments from this national programme designed to reduce (rather than exacerbate) inequalities. Moreover, as observed here, there were significant differences in B4SC uptake between Pacific ethnicities. An additional important consideration was the 16.1% of Pacific children within the IDI dataset that did not appear enrolled within the MoE database due to delayed school commencement, home-schooling, emigration outside of New Zealand, or unsuccessful linking. Relatively small but significant differences were observed between those within and outside the MoE databases which may affect the external validity of the study findings. Further, it must be noted that the analyses are hinged on routinely collected data that are inputted from multiple individuals in various locations over time. As such, operationalised variations in exposure and classification definitions potentially exist or evolve that may differentially comprise the internal validity of any findings.

The study raises a number of possible policy and practice outcomes. Generally it is cited that a key policy lever is lowering cost barriers to make primary health care more accessible for all people^54^. But to increase Pacific engagement this, by itself, is not sufficient. To address the issues associated with the cultural acceptability of services, institutional racism, language barriers, and socioeconomic deprivation, there is a need for partnerships in the planning and delivery of services; the shared value of services delivered in churches and in locations where Pacific feel comfortable, at times which allow flexibility; and the benefits of access to Pacific health and education professionals and community workers^55^. There is also the need for structural change in the provision of health and educational services if they are to be more responsive to the needs of Pacific people. Developing a culturally responsive service may involve working in a different way that takes much greater account of the values, beliefs and practices of Pacific people.

In conclusion, Pacific children in New Zealand are ethnically and culturally diverse. Large ethnic differences existed in the likelihood of receiving literacy interventions between these Pacific children. Equity demands that we address the relatively poor literacy achievement and needs of Pacific children. However, it is important that researchers and policy-makers consider the question of “which children,” as the literacy achievement gap is far from uniform across the sociodemographic and health indicator profile of Pacific children considered here. Once identified, then efficacious culturally-nuanced strategies might be implemented to help ameliorate this disparity. Predictively, the utilised demographic and B4SC failed to produce a model of reasonable power, and this begs further continued research. But unless we are content to continuing leaving Pacific children behind in New Zealand, do we have enough evidence to begin answering the “which children” question?

## Methods

### Study design

Time-to-event analysis of a continuously recruited national cohort.

### Participants

Children identified as being of at least one Pacific ethnicity, born between 1 January 2005 and 31 December 2011, inclusive, who were in the MoH (B4SC) and/or MoE databases and captured within the IDI. Ethnicity was based on parental/caregiver report and allowed multiple identities, as is the prevailing method of reporting in New Zealand^17^.

### Outcome measure: literacy intervention

New Zealand children typically start formal schooling around their 5^th^ birthday; although it is only at their 6^th^ birthday that they are legally obligated to commence. After approximately one school year of classroom instruction, children are assessed on a range of literacy and language measures. Those falling behind their peers in early reading development are identified for reading intervention. Data were gleaned from the MoE reports contained within the IDI, which captures each instance where a child has received a school-based intervention. Literacy intervention was assessed here via: ‘Reading recovery’, which is an intensive reading program targeting children aged 6 years, typically having completed their first year of primary schooling; and ‘Specialist teaching’, whereby specialist resource teachers’ work with students who are struggling to meet national literacy standards for their age. Although nationally administered, around 72% of all children aged 6 years have potential access to the ‘Reading recovery’ intervention as some schools choose not to implement it^36^. A binary ‘literacy intervention’ variable was created, and indicated if either ‘Reading recovery’ or ‘Specialist teaching’ interventions was initiated, together with the child’s age that this first occurred. Children without either intervention by the study end-date were treated as censored. A study end-date of 31 December 2015 was applied to ensure record completeness within the IDI.

### Ethnicity, demographic and B4SC explanatory variables

Within New Zealand, ethnicity is classified in level hierarchies, with level I information identifications grouped as: European; Māori; Pacific; Asian; Middle Eastern/Latin American/African; and, Other Ethnicity. For parents/caregivers with Pacific children, they can further identify their child (level II information) as belonging to one or more of the following Pacific ethnic groups: Samoan; Cook Islands Māori; Tongan; Niuean; Tokelauan; Fijian; other Pacific peoples; and, Pacific peoples not defined. Children’s ethnicity was ascribed from SNZ Census, Department of Internal Affairs - Te Tari Taiwhenua (DIA; Births, Deaths and Marriages Register), and MoH sources using a priority approach. If ethnic identifications were level I or missing from the SNZ Census data file, then the DIA values were used if they contained level II information; and if ethnic identifications were level I or missing from SNZ Census and DIA files, then the MoH information was utilised if they contained level II information. Due to the known demographic profile^16^, an *a priori* decision was made to combine the level II ethnic groups of Niuean; Tokelauan; Fijian; other Pacific peoples; and, Pacific peoples not defined into an ‘Other Pacific’ group.

As multiple ethnicities can be indicated, this implies that New Zealand’s ethnic categories are not mutually exclusive, making direct comparison of individual ethnic groups difficult. In an effort to improve interpretability, mutually exclusive major ethnic group combinations were developed, based on the population priorities within New Zealand (Māori being the indigenous people of New Zealand; and Europeans representing the dominant population group) and on their empirical distributions. As such, eight mutually exclusive categories were defined: Samoan; Cook Islands Māori; Tongan; Other Pacific (which also included those of two or more Pacific ethnicities); Pacific and Māori; Pacific and European; Pacific and Māori and European; and, Pacific and Other. The last groups includes those children identified as being of Asian, Middle Eastern, Latin American, African, or other ethnicity.

Sex was categorised as female (girls) and male (boys). Age (in months) was calculated from month/year of birth. Area level deprivation was measured using the New Zealand Index of Deprivation 2013 (NZDep2013)^37^ for the recorded residential address of the child at their B4SC. It is based on the deprivation characteristics of “meshblocks” (small areas with a typical population of 60–110 people), and combines 2013 Census data relating to income, home ownership, employment, qualifications, family structure, housing, access to transport and communications into a single measure. Each meshblock is assigned a score from 1 (least deprived) to 10 (most deprived), with 10% of all meshblocks being in each category. While deprivation is categorised in deciles, quintiles were employed here. Urban/rural residency was derived from the New Zealand standard classification which uses a 5-point scale, namely: (i) main urban (centred on a city or major urban area with population of 30,000+ people); (ii) secondary urban (centred on larger regional centres with population of 10,000–29,999 people); (iii) minor urban (centred around smaller towns with population of 1,000–9,999 people); (iv) rural centre (with population of 300–999 people); and (v) other rural (inlets, islands, inland waters, and oceanic waters)^56^. These were collapsed into two groups: urban, combining (i)–(iii); and, rural, combining (iv)–(v).

The B4SC includes measures of hearing and vision, height and weight, and behavioural and developmental questions using the SDQ and PEDS tools. Detailed information on the measurement and reporting of these variables can be found elsewhere^21,24^, and are summarised in Table 3.

**Table 3:** B4SC variables utilised and their definition.

Variable	Definition
*Hearing*:	Audiometry initially uses the sweep test to screen for asymptomatic hearing loss. The procedure is based on the 1997 American Speech-Language-Hearing Association screening guidelines^59^. The technique relies on a conditioned response to sound. If the sweep test result is normal, no further tests are undertaken and the results recorded. If the sweep test result is equivocal or abnormal, tympanometry is undertaken and a clinical referral pathway invoked and recorded^21^. Audiometry screening is unnecessary for some children (i.e., if under the care of an otorhinolaryngology specialist or an audiologist, wears a hearing aid, or has a cochlear implant or grommets); these reasons are noted in the child’s B4SC record.
*Vision*:	The most commonly used acuity screen is one containing lines of letters (e.g., the Snellen vision test) or one in which single letters have neighbouring ‘confusion bars’ (e.g., Parr letter-matching test or the equivalent Sheridan Gardner charts test)^21^. If the child’s vision is: 6/9 or better in both eyes then this is considered a pass, their results recorded and no further action taken; 6/9 in one eye and 6/6 in the other then the measurements are recorded and a rescreen arranged (3–6 months later); 6/12 or worse in either eye or both eyes, then this is considered a fail and referral for an ophthalmic assessment according to local protocols invoked and recorded. On rescreening, if there is no change or worsening in either eye, then this is considered a fail and referral invoked and recorded^21^.
*Height, weight and body mass index (BMI)*:	Anthropometric measurements were undertaken by registered nurses or nurse practitioners, who received a handbook outlining best-practice protocols^21^, including measuring the children while they were wearing light clothing with shoes removed, with the equipment stable on a levelled hard surface. Height was to be measured to the nearest 0.1 cm using a portable stadiometer (either Leicester Height Measure or a SECA 214) and weight to the nearest 0.1 kg using a SECA 862 electronic floor scale or Tanita WB 100 S MA floor scale (or SECA 770 or Tanita HD-351 weighing scale; calibrated at least once every 6 months)^22^. Body mass index (BMI) was then derived by dividing each child’s weight by their height squared (i.e. kg/m^2^). The World Health Organization (WHO) Anthro (version 3.2.2) macro for Stata was used to derive WHO growth standard measures that yielded sex-specific BMI-for-age z-scores^22,60^. Children at, or above, the 85th, 95th, and 99.7th percentile for sex and age adjusted BMI were classified as overweight, obese, and extremely obese, respectively^22^.
*Strengths and Difficulties Questionnaire (SDQ)*:	Evaluates children’s emotional and behavioural development over the last six months^61^. It is comprised of five scales, each having five items that asks about the child’s psychosocial attributes (positive and negative behaviours): emotional attributes, conduct, hyperactivity, peer relations and prosocial behaviour. It also asks about how the child’s behavioural difficulties affect the child’s life. The SDQ score is significantly more sensitive if both a parent/care-giver and teacher complete the questionnaire^62^. Therefore, if a child is involved in early childhood education, his/her teacher is encouraged to complete the teacher version of the questionnaire and asked to discuss their findings with the child’s parents. The parent/caregiver and teacher complete the questionnaire by marking each item as “Not true”, “Somewhat true” or “Certainly true”. Reponses are weighted and yield a score between 0 and 10 for each scale, together with a total difficulties score of between 0 and 40. Scores are classified into ‘normal’, ‘borderline’ or ‘abnormal’ groups for each attribute and for the total difficulties score. Depending on the pattern of scores from parents/care-givers and/or teachers, five clinical referral pathways are available: (i) refer child to paediatrician of Children and Adolescent Mental Health Services; (ii) refer child to Group Special Education; (ii) refer child to Strengthening Families; (iv) manage child in service; and (v) no further action required. The SDQ, its scoring and interpretation is reproduced with permission, and clinical referral pathways discussed in detail elsewhere^21^.
*Parents’ Evaluation of Developmental Status (PEDS)*:	A questionnaire for parents to detect developmental and behavioural problems in children from birth to eight years^48^. The PEDS has 10 general questions about behaviour, development, speech and language, and fine and gross motor skills (e.g., ‘Do you have any concerns about how the child talks and makes speech sounds?’ With response options: “No”, “Yes”, and “A little” and a free-text “Comments” option). Parents fill in the questionnaire, which also includes guidelines. Based on the pattern of response, one of five pathways are invoked: (i) two or more significant concerns: (a) about self-help, social, school, or receptive language skills then refer for audiological and speech-language testing, and use professional judgment to decide if referrals are also needed for social work, occupational/physiotherapy, mental health services, etc. or (b) for other concerns then refer for intellectual and educational assessments, and use professional judgment to decide if speech-language, audiological, or other evaluations are also needed; (ii) one significant concern – a secondary screen is needed; (iii) non-significant concern – counsel the parent in the area of difficulty and arrange follow up if necessary; (iv) parental communication difficulty – may need an interpreter or to consider a different screen; and (v) no concerns – no further action required. The PEDS, its scoring form and interpretation form are reproduced with permission elsewhere^21^.

### Procedure

Presentation of methods and reporting of findings were informed by the STROBE guidelines (www.strobe-statement.org). A thorough description of the procedures for the implementation of both the B4SC and literacy intervention have also been provided elsewhere^21,24^. In brief, B4SC are conducted by registered nurses and hearing and vision technicians. If concerns are identified, the child and their parents or caregivers are offered information and support which include clinical pathways and referral processes to health, education or social support services. B4SCs are undertaken in various locations, depending on the needs of the community. After receiving informed parental/caregiver informed consent, they usually takes 45–60 minutes to complete^21^. Held by the MoH, the B4SC National Information System stores data relating to the child, permission, assessments and checks, and any issues identified and referrals made. This system provides non-identifiable information for monitoring the performance of the B4SC programme, for tracking the population health status of 4-year-olds, and for approved research studies^21^. The MoE routinely collects intervention data on all children enrolled within its service such as intervention type, date and timing, frequency and duration. As part of the IDI scope and mandate, these data are digested into the infrastructure. These apposite data from the MoH and MoE databases were linked through the IDI platform, together with augmented data from SNZ (Census 2013) and DIA. Conducted by SNZ, the IDI uses both deterministic and probabilistic techniques to link individuals across datafiles using a unique identifier that is derived from an individual’s name, date of birth, sex, and home address^22^. Missing information for a variable in one database may be replenished by non-missing data for a duplicate variable from another source. If conflicting information existed in duplicate fields (such as ethnic identification), then Census data were preferred, followed by DIA and then MoH data. All analyses are performed in a secured environment, according to strict protocols, and findings released after SNZ IDI approval^19^.

### Statistical analysis

Analyses and graphs were performed using Stata MP version 14.0 (StataCorp, College Station, TX, USA), and α = 0.05 defined statistical significance. Initially, demographic distributions for selected variables were derived and compared between the full IDI database and those within the MoE sample. The empirical overall distribution of children’s first literacy intervention, their median age and associated quartiles, together with the distributions by selected demographics and the Kaplan-Meier curves by ethnicity, sex and deprivation were given. Unadjusted and adjusted Cox proportional hazard models using a robust estimator of variance predicting the likelihood of children’s first literacy intervention were then implemented on these selected demographic variables and the considered B4SC variables, with HRs and associated 95% CIs reported (employing the ‘stcox’ command in Stata). Note, because of children’s different follow-up times and the right censoring of many participants, considerable care needs to be exercised in interpreting and comparing the percentages (%) of children who were observed to have a literacy intervention and the associated estimated risks (derived from the Kaplan-Meier curves and Cox proportional hazard models). Due to the almost complete concordance of ‘declined test’ and ‘not done (missing)’ values between hearing and vision tests, and for ‘not done (missing)’ values of the PEDS, SDQ-parent or caregiver, and SDQ-teacher variables, only one apposite indicator variable was used in each variable grouping for the adjusted analyses. For instance, in reporting the adjusted HR and 95% CI for hearing, the ‘declined test’ and ‘not done (missing)’ indicator variables were included for hearing but omitted for vision, whereas in reporting the adjusted HR and 95% CI for vision, the ‘declined test’ and ‘not done (missing)’ indicator variables for vision were employed but those associated with hearing were omitted. The ‘lincom’ command in Stata was used to derive the point estimate, standard error, significance, and 95% CI for linear combinations of risk factors. To assess the contribution of the B4SC variables in increasing the model’s predictive power over and above the adjusted model containing only the selected demographic variables, Harrell’s *c*-statistic was employed^57^. The *c*-statistic gives the probability a randomly selected participant who experienced an event (e.g. the literacy intervention) had a higher risk score than a participant who had not experienced the event. A value of 0.5 indicates that the model is no better than chance at making a prediction and a value of 1.0 indicates that the model perfectly identifies those within a group and those not. Models are typically considered reasonable when the *c*-statistic is higher than 0.7 and strong when it exceeds 0.8^51^. Following the method advocated by Newson, the dataset was randomly partitioned into two; the first used as a training dataset to fit the model, and the second used as a test dataset to make prediction assessments^57^. Agreement between the SDQ-parent/caregiver and SDQ-teacher scores were assessed using the κ statistic. Using Landis and Koch’s characterization, κ > 0.75 was taken to represent strong agreement, 0.40 ≤ κ ≤ 0.75 was taken to represent moderate agreement, and κ < 0.40 was taken to represent poor agreement^58^.

### Approvals and ethics

The study proposal and protocols were approved by SNZ (MAA2017-15) and by the University of Otago Human Ethics Committee (D17/024). Based on New Zealand’s Health and Disability Ethics Committees’ checklist, the study did not meet the threshold required for formal ethics review. All methods and reported results were carried out in accordance with relevant guidelines and regulations, and only includes aggregated randomly rounded to base 3 de-identified data.

### Data availability

The datasets used for statistical analysis are held within the IDI. Application to use these data must be made through SNZ.

### Disclaimer

The results in this paper are not official statistics. They have been created for research purposes from the IDI, managed by SNZ. The opinions, findings, recommendations, and conclusions expressed in this paper are those of the authors, not SNZ. Access to the anonymised data used in this study was provided by SNZ under the security and confidentiality provisions of the Statistics Act 1975. Only people authorised by the Statistics Act 1975 are allowed to see data about a particular person, household, business, or organisation, and the results in this paper have been confidentialised to protect these groups from identification and to keep their data safe. Careful consideration has been given to the privacy, security, and confidentiality issues associated with using administrative and survey data in the IDI. Further detail can be found in the privacy impact assessment for the IDI available from www.stats.govt.nz.

## References

[1] Shaw A, Canadian Paediatric Society (2006). Read, speak, sing: promoting literacy in the physician’s office. Paediatr Child Health.

[2] D’Eath, M., Barry, M. M. & Sixsmith, J. Rapid evidence review of interventions for improving health literacy. (European Centre for Disease Prevention and Control, Stockholm, 2012).

[3] Santo A, Laizner AM, Shohet L (2005). Exploring the value of audiotapes for health literacy: a systematic review. Patient Educ Couns.

[4] Abrams MA, Klass P, Dreyer BP (2009). Health literacy and children: recommendations for action. Pediatrics.

[5] Neumann MM (2016). A socioeconomic comparison of emergent literacy and home literacy in Australian preschoolers. Eur Early Child Educ Res J.

[6] Gillon, G. *Phonological awareness: from research to practice*. 2nd edn (Guilford Press, 2017).

[7] Hart, B. & Risely, T. R. *Meaningful differences in the everyday experience of young American children* (Paul Brookes Publishing Company, 1995).

[8] Statistics New Zealand - Tatauranga Aotearoa (SNZ). 2013 Census - Major ethnic groups in New Zealand (SNZ, Wellington, 2018).

[9] Statistics New Zealand - Tatauranga Aotearoa (SNZ). Pacific progress: a report on the economic status of Pacific peoples in New Zealand (SNZ, Wellington, 2002).

[10] Ministry of Health - Manatū Hauora (MoH). Tupu Ola Moui: Pacific Health Chart Book 2012 (MoH, Wellington, 2012).

[11] Ministry of Health - Manatū Hauora (MoH). Pacific Child Health: a paper for the Pacific Health and Disability Action Plan review (MoH, Wellington, 2008).

[12] Taleni, T. *‘**E saili i tautai se agava’a - A true leader masters the art of navigation’. The impact of effective leadership in raising engagement and achievement of Pasifika learners in New Zealand schools* Master of Education thesis, University of Canterbury (2017).

[13] Ministry of Education - Te Tāhuhu o te Mātauranga (MoE). Pasifika Education Plan 2013–2017 (MoE, Wellington, 2013).

[14] Ministry of Education - Te Tāhuhu o te Mātauranga (MoE). Tapasā draft framework consultation feedback summary report (MoE, Wellington, 2017).

[15] Borrows J, Williams M, Schluter P, Paterson J, Helu SL (2011). Pacific Islands Families Study: the association of infant health risk indicators and acculturation of Pacific Island mothers living in New Zealand. J Cross Cult Psychol.

[16] Ministry for Pacific Peoples - Te Manatū mō Ngā Iwi o Te Moana-nui-a-Kiwa (MPP). Pacific People in N*Z*, http://www.mpp.govt.nz/pacific-people-in-nz (2017).

[17] Statistics New Zealand - Tatauranga Aotearoa (SNZ). Classification of ethnicity, http://archive.stats.govt.nz/methods/classifications-and-standards/classification-related-stats-standards/ethnicity.aspx (2005).

[18] Statistics New Zealand - Tatauranga Aotearoa (SNZ). 2013 Census QuickStats about culture and identity, http://www.nbr.co.nz/sites/default/files/quickstats-culture-identity.pdf (2014).

[19] Statistics New Zealand - Tatauranga Aotearoa (SNZ). Integrated Data Infrastructure, https://www.stats.govt.nz/integrated-data/integrated-data-infrastructure/ (2017).

[20] Ministry of Health - Manatū Hauora (MoH). B4 School Check, https://www.health.govt.nz/our-work/life-stages/child-health/b4-school-check (2015).

[21] Ministry of Health - Manatū Hauora (MoH). The B4 School Check: a handbook for practitioners (MoH, Wellington, 2008).

[22] Shackleton, N. *et al*. Improving rates of overweight, obesity and extreme obesity in New Zealand 4-year-old children in 2010–2016. *Pediatr Obes*, 10.1111/ijpo.12260 (2017). 10.1111/ijpo.12260 PMC6585814 29271074

[23] Ministry of Education – te Tāhuhu o te Mātauranga (MoE). Our purpose, vision and behaviours, https://education.govt.nz/ministry-of-education/our-role-and-our-people/our-purpose-vision-and-behaviours/ (2015).

[24] Schluter, P. J. *et al*. The efficacy of preschool developmental indicators as a screen for early primary school-based literacy interventions. *Child Dev*, under review (2018). 10.1111/cdev.13145 30204249

[25] Anderson LM (2003). The effectiveness of early childhood development programs. A systematic review. Am J Prev Med.

[26] Willis E, Kabler-Babbitt C, Zuckerman B (2007). Early literacy interventions: reach out and read. Pediatr Clin North Am.

[27] Zubrick SR, Taylor CL, Christensen D (2015). Patterns and predictors of language and literacy abilities 4-10 years in the Longitudinal Study of Australian Children. PLoS One.

[28] Christensen D, Zubrick SR, Lawrence D, Mitrou F, Taylor CL (2014). Risk factors for low receptive vocabulary abilities in the preschool and early school years in the longitudinal study of Australian children. PLoS One.

[29] Reilly S (2010). Predicting language outcomes at 4 years of age: findings from Early Language in Victoria Study. Pediatrics.

[30] Duff FJ, Reen G, Plunkett K, Nation K (2015). Do infant vocabulary skills predict school-age language and literacy outcomes?. J Child Psychol Psychiatry.

[31] Statistics New Zealand - Tatauranga Aotearoa (SNZ). 2013 Census tables, http://nzdotstat.stats.govt.nz/wbos/Index.aspx (2015).

[32] Han WJ, Huang CC (2010). The forgotten treasure: bilingualism and Asian children’s emotional and behavioral health. Am J Public Health.

[33] Han WJ (2012). Bilingualism and academic achievement. Child Dev.

[34] Disenhaus, N. Boys, writing, and the literacy gender gap: what we know, what we think we know (University of Vermont, Burlington, VT, 2015).

[35] Moss, G. & Washbrook, L. Understanding the gender gap in literacy and language development. Bristol Working Papers in Education #01/2016 (University of Bristol, Bristol, 2016).

[36] Ministry of Education – te Tāhuhu o te Mātauranga (MoE). Annual monitoring of Reading Recovery, https://www.educationcounts.govt.nz/publications/series/1547 (2017).

[37] Atkinson, J., Salmond, C. & Crampton, P. NZDep2013 Index of Deprivation (Department of Public Health, University of Otago, Wellington, 2014).

[38] Simpson, J., Duncanson, M., Oben, G., Wicken, A. & Gallagher, S. Child Poverty Monitor Technical Report 2016 (New Zealand Child and Youth Epidemiology Service, University of Otago, Dunedin, 2016).

[39] Solari CD, Mare RD (2012). Housing crowding effects on children’s wellbeing. Soc Sci Res.

[40] Datar A, Sturm R, Magnabosco JL (2004). Childhood overweight and academic performance: national study of kindergartners and first-graders. Obes Res.

[41] Martin A (2017). Longitudinal associations between childhood obesity and academic achievement: systematic review with focus group data. Curr Obes Rep.

[42] Vaz S (2016). Is using the Strengths and Difficulties Questionnaire in a community sample the optimal way to assess mental health functioning?. PLoS One.

[43] Fortnum H (2016). A programme of studies including assessment of diagnostic accuracy of school hearing screening tests and a cost-effectiveness model of school entry hearing screening programmes. Health Technol Assess.

[44] Schluter PJ, Bridgford P, Cook L, Hamilton G (2014). Improving the evidence-base for access to primary health care in Canterbury: a panel study. Aust N Z J Public Health.

[45] Paterson J (2006). Pacific Islands Families: First Two Years of Life Study - design and methodology. N Z Med J.

[46] Paterson J (2008). Cohort profile: The Pacific Islands Families (PIF) Study. Int J Epidemiol.

[47] Paterson JE (2006). Pacific Islands families study: the prevalence of chronic middle ear disease in 2-year-old Pacific children living in New Zealand. Int J Pediatr Otorhinolaryngol.

[48] Glascoe FP (2003). Parents’ evaluation of developmental status: how well do parents’ concerns identify children with behavioral and emotional problems?. Clin Pediatr.

[49] Harrison LJ, McLeod S, Berthelsen D, Walker S (2009). Literacy, numeracy and learning in school-aged children identified as having speech and language impairment in early childhood. Int J Speech Lang Pathol.

[50] Wake M, Gerner B, Gallagher S (2005). Does parents’ evaluation of developmental status at school entry predict language, achievement, and quality of life 2 years later?. Ambul Pediatr.

[51] Hosmer, D. W. & Lemeshow, S. Applied Logistic Regression. 2nd edn (John Wiley & Sons, New York, NY, 2000).

[52] Shmueli G (2010). To explain or to predict?. Stat Sci.

[53] Litmus. Well Child Tamariki Ora Programme Quality Reviews (Limus, Wellington, 2013).

[54] Jatrana S, Crampton P (2009). Primary health care in New Zealand: who has access?. Health Policy.

[55] Barwick, H. Improving Access to Primary Care for Māori and Pacific Peoples: A Literature Review Commissioned by the Health Funding Authority (Health Funding Authority, Wellington, 2000).

[56] Statistics New Zealand – Tatauranga Aotearoa (SNZ). Classifications and related statistical standards: Urban Area. Secondary classifications and related statistical standards: Urban Area, http://archive.stats.govt.nz/methods/classifications-and-standards/classification-related-stats-standards/urban-area.aspx (2016).

[57] Newson BR (2010). Comparing the predictive powers of survival models using Harrell’s C or Somers’ D. Stata J.

[58] Landis JR, Koch GG (1977). The measurement of observer agreement for categorical data. Biometrics.

[59] American Speech-Language-Hearing Association (ASHA). Guidelines for audiologic screening (ASHA, Rockville, MD, 1997).

[60] World Health Organization (WHO). Child growth standards. WHO Anthro (version 3.2.2, January 2011) and macros, http://www.who.int/childgrowth/software/en (2016).

[61] Goodman R (1997). The Strengths and Difficulties Questionnaire: a research note. J Child Psychol Psychiatry.

[62] Goodman R, Ford T, Simmons H, Gatward R, Meltzer H (2000). Using the Strengths and Difficulties Questionnaire (SDQ) to screen for child psychiatric disorders in a community sample. Br J Psychiatry.

